# New bactericide derived from Isatin for treating oilfield reinjection water

**DOI:** 10.1186/1752-153X-6-90

**Published:** 2012-08-28

**Authors:** Gang Chen, Hui-jun Su, Min Zhang, Fang Huo, Jie Zhang, Xiao-jiang Hao, Jing-rui Zhao

**Affiliations:** 1 College of Chemistry and Chemical Engineering, Xi’an Shiyou University, Xi’an, 710065, People’s Republic of China; 2 State Key Laboratory of Phytochemistry and Plant Resources in West China, Kunming Institute of Botany, Chinese Academy of Sciences, Kunming, 650204, People’s Republic of China; 3 Shannxi Hai’an Industry Co., LTD, Xi’an, 710065, People’s Republic of China

## Abstract

Isatin, an extract from *Strobilanthes cusia* (Nees) Kuntze, was the base for synthesizing derivatives that were screened for antibacterial activity against oilfield water-borne bacteria. The bacterial groups are sulfate reducing, iron and total. The derivatives were characterized by spectrums and they showed good to moderate activity against sulfate reducing bacteria.

## Background

The roots and the leaves of the plant, *Strobilanthes cusia* (Nees) Kuntze of the Acanthaceae family that is widely distributed in northern and central China, have been used in traditional Chinese medicine to treat a variety of ailments caused by microorganisms and virus. It is suggested that the demonstrated use can be extended to processing oilfield water to remove or reduce bacteria before the water is re-injected into formations via wells.

**Figure 1:**
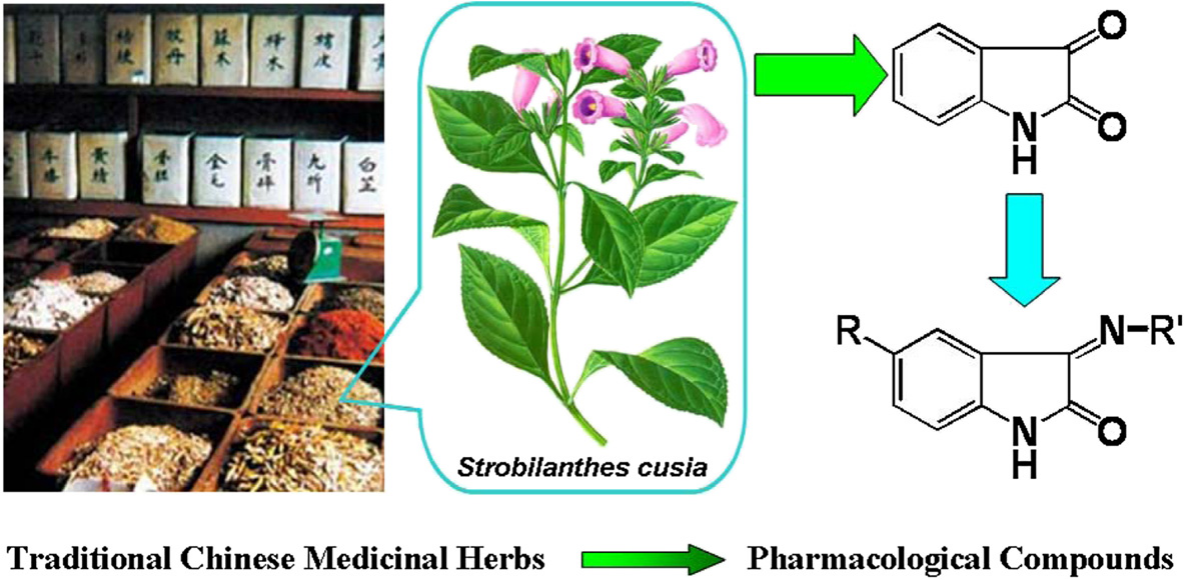
Development of new bactericide for oilfield reinjection water treatment from traditional Chinese medicine.

The alkaloid isatin or indole-2-3-dione (Figure 1) is a compound found in *Strobilanthes cusia* (Nees) Kuntze and many other plants such as genus *Isatis*, *Calanthe discolor* LINDL, *Couroupita guianensis Aubl.* and in mammalian tissue [1]. It has versatile bioactivity [2] and it is used to synthesize a large variety of heterocyclic compounds in preparing drugs [3-7]. Isatin Schiff bases are reported to have antibacterial activity against *Bacillus subtilis*[8], Gram(+) and Gram(−) bacterial strains [9] and *Magnaporthe grisea*[10] among others. The compound has been produced industrially and can thus be used for large-scale applications such as treating oilfield water before re-injection.

## Base synthesis and identification

### Synthesis of isatin derivatives

Isatin (1 mmol) was dissolved in methanol (20 ml) and a methanol solution of 1.2 mmol amino compound (10 ml) was added dropwise, until the disappearance of isatin, as evidenced by thin-layer chromatography. The solvent was removed in vacuo and the residue was separated by column chromatography (silica gel, petroleum ether/ ethyl acetate = 1:1 ~ 1:3 v/v), to give the product. Single crystals of the compound 4 suitable for X-ray analysis was obtained on slow evaporation of a methanol solution (30 ml) of the product (30 mg) over a period of 7 d.

### X-ray data collection and structure refinement

Intensity data for colorless crystals of compound 4 was collected at 150 K on a Bruker SMART 1000 CCD fitted with Mo Ka radiation. The data sets were corrected for absorption based on multiple scans [11] and reduced using standard methods [12]. The structures was solved by direct-methods [13] and refined by a full-matrix leastsquares procedure on F^2^ with anisotropic displacement parameters for non-hydrogen atoms, carbon-and nitrogen bound hydrogen atoms in their calculated positions and a weighting scheme of the form *w* = 1/*σ*^2^(*F*_o_^2^ ) + (*αP*)^2^ + *bP* where *P* = (*F*_o_^2^ + 2*F*_c_^2^)/3) [14]. Crystal data and refinement details were given in Table 1.

**Table 1:** Experimental data of compound A and B

**Empirical formula**	**C**_**8**_**H**_**6**_**N**_**2**_**O**_**2**_
Formula weight	162.15
Temperature	293(2)
Wavelength (Mo K_α_)	0.71073
Crystal system	Monoclinic
Space group	*P2(1)*
Crystal data	
a (Ǻ)	3.8800(6)
b (Ǻ)	10.180(9)
c (Ǻ)	9.0500(6)
α (º)	90.00
β (º)	93.90(3)
γ (º)	90.00
Volume	356.63(12)
Z	2
Density (mg/m^3^)	1.510
Absorption coefficient	0.112
F (000)	168
Crystal size	0.20 × 0.22 × 0.30 mm^3^
Theta range for data collection (°)	1.9 to 27.3
Index ranges	−4 ≤ h ≤ 5; −13 ≤ k ≤ 12; −11 ≤ l ≤ 12
Reflections collected	3112
Independent reflections	2190
Reflections theta (°)	2.26 to 28.27
Absorption correction transmission	0.9440 to 0.9861
Reflections with I ≥ 2σ(*I*)	1427
Number of parameters	109
Goodness-of-fit on F^2^	1.006
Final R indices [I>2 s(I)]	R1 = 0.1884; wR2 = 0.1323
R indices (all data)	R1 = 0.0674; wR2 = 0.1027
Refine different density	−0.224 to 0.176

### Microbiological monitoring

Viable counts of SRB, TGB and FB were determined with the “most probable number” method, People’s Republic of China Standard of Petroleum and Natural Gas Industry, the national method of the bactericidal agent’s performance, SY/T 5890–1993). The produced water containing the three kinds of bacteria was gathered from Zichang Oilfield Factory, Yanchang Oilfield.

## Results and discussion

### Chemistry

**Scheme 1:**
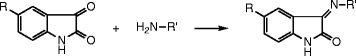
Synthesis of isatin derivatives by condensation reaction.

The isatin derivatives were synthesized as shown in Scheme 1. All the isatin derivatives were characterized by ^1^ H-NMR (400 MHz) and MS (EI) spectra and the results were summarized in Table 2. The entire spectra consist with the anticipated structures.

**Table 2:** **The**^**1**^ **H-NMR (400 MHz) and MS (EI) spectra of the isatin derivatives**

**Comp. no.**	**Structure**	^1^** H-NMR (400 MHz) and MS (EI) spectra**
1		/
2		/
3		/
4		^1^ H-NMR (D_6_-Acetone), δ: 8.05 (1 H, d, *J =*7.2 Hz), 7.36 (1 H, t, *J =* 7.6 Hz), 7.04 (1 H, t, *J =* 7.6 Hz), 6.95 (1 H, d, *J =* 7.6 Hz), 6.84 (1 H, d, *J =* 7.6 Hz); MS *m/z*: 162 (M^+^)
5		^1^ H-NMR (D_6_-DMSO), δ: 12.40 (1 H, s), 11.08 (1 H, s), 9.06 (1 H, s), 8.95 (1 H, s), 7.66 (1 H, d, *J =* 7.6 Hz), 7.30 (1 H, t, *J =* 7.6), 7.10 (1 H, t, *J =* 7.6 Hz), 6.90 (1 H, d, *J =* 8.0 Hz); MS *m/z*: 204 (M^+^)
6		^1^ H-NMR (D_6_-DMSO), δ: 12.46 (1 H, s), 11.02 (1 H, s), 9.05 (1 H, s), 8.96 (1 H, s), 7.64 (1 H, d, *J =* 7.6 Hz), 7.34 (1 H, t, *J =* 7.6), 7.08 (1 H, t, *J =* 7.6 Hz), 6.91 (1 H, d, *J =* 8.0 Hz); MS *m/z*: 220 (M^+^)
7		^1^ H-NMR (D_6_-DMSO), δ: 12.01 (1 H, s), 11.01 (1 H, s), 9.23 (1 H, s), 8.90 (1 H, s), 7.54 (1 H, d, *J =* 7.6 Hz), 7.30 (1 H, t, *J =* 7.6), 6.86 (1 H, d, *J =* 8.0 Hz), 2.48 (1 H, s); MS *m/z*: 218 (M^+^)
8		^1^ H-NMR (D_6_-DMSO), δ: 10.92 (1 H, s), 9.56 (1 H, s), 7.32 (2 H, m), 6.86 (4 H, m), 6.74 (3 H, m); MS *m/z*: 238 (M^+^)
9		^1^ H-NMR (CDCl_3_), δ: 9.14 (1 H, s), 7.32 (1 H, d, *J =* 7.2 Hz), 7.08 (2 H, d, *J =* 8.8 Hz), 6.99 (3 H, m), 6.93 (1 H, d, *J =* 8.0 Hz), 6.79 (1 H, t, *J =* 7.6 Hz), 3.88 (3 H, s); MS *m/z*: 253 (M^+^)
10		^1^ H-NMR (CDCl_3_), δ: 9.98 (1 H, s), 7.64 (1 H, d, *J =* 7.2 Hz), 7.42 (1 H, d, *J =* 7.2 Hz), 7.14 (2 H, d, *J =* 8.8 Hz), 7.02 (3 H, m), 6.81 (1 H, d, *J =* 8.0 Hz); MS *m/z*: 256 (M^+^)

**Figure 2:**
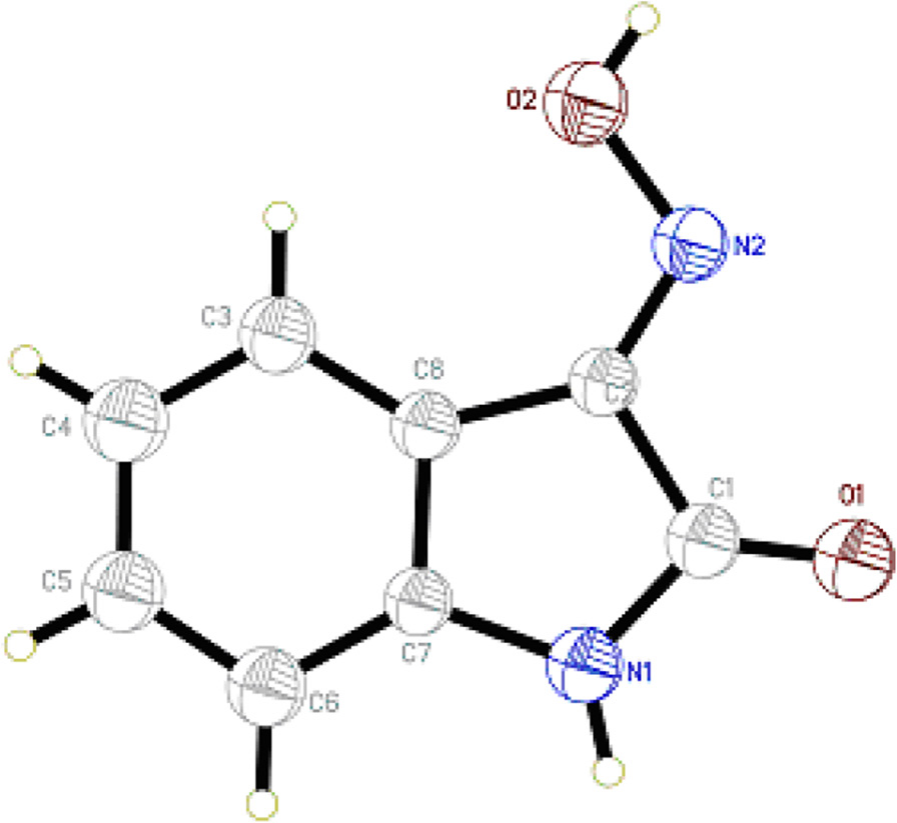
An ORTEP-3 drawing of compound 4, with the atom-numbering scheme and 30% probability displacement ellipsoids.

**Figure 3:**
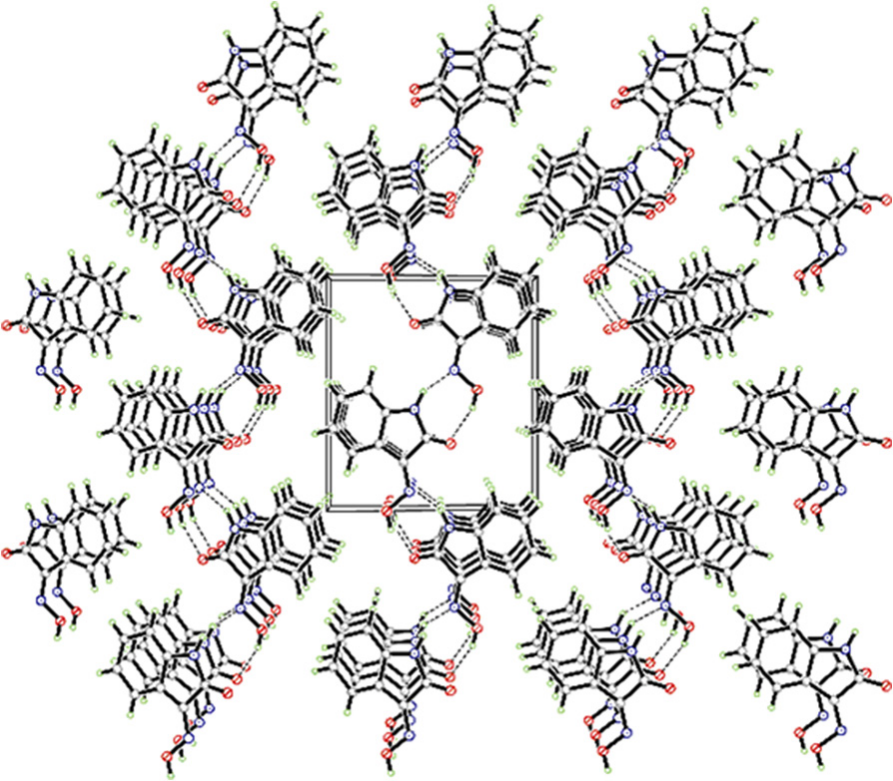
Packing of compound 4, dashed lines indicating hydrogen bonds.

Besides, single crystal of compound 4 was analysized by X-ray, which confirms the assignment of the structure from spectroscopic data. The values of the geometric parameters of compound 4 are within normal ranges and experimental errors. The X-ray structural analysis confirmed the assignment of its structure from spectroscopic data. The molecular structure is depicted in Figure 2, and a packing diagram of compound 4 is depicted in Figure 3. Geometric parameters of compound 4 are in the usual ranges. The indol-2-one ring system is substantially planar. In the crystal structure, intermolecular N—H—N and O—H—O hydrogen bonds (Table 3) are effective in the stabilization of the structure and are responsible for the formation of a one-dimensional network. The angle of C1—C2—N2 is 115.734°, and the angle of C2—N2—O2 is 112.199°.

**Table 3:** Hydrogen-bond geometry in the crystal of compound 4 (Å, °)

**D—H···A**	**D—H**	**H···A**	**D···A**	**D—H···A**
N1—H1A···N2i	0cxx.86	2.10	2.831 (8)	142
O2—H2A···O1ii	0.82	1.92	2.698 (5)	159

*Symmetry codes: (i) − x + 1, y + 1/2, −z + 2; (ii) − x + 1, y − 1/2, −z + 2.

### Bioactivity

Produced water is a consequence of an oilfield exploitation that uses waterflood or steam injection or has an aquifer linked to the reservoir. The most usual disposal ways for high volumes of produced water is re-injected after treatment, which will meet some requirements imposed by environmental regulations [15]. Microbiologically influenced corrosion (MIC) caused by growth of sulfate reducing bacteria (SRB), iron bacteria (IB) and total general bacteria (TGB) in oil pipelines, is considered a major problem for water treatment in the oil industry [16]. MIC can result in different types of attack: pitting, crevices, dealloying and erosion in pipelines [17]. Corrosion products produced by microorganisms are production of hydrogen sulfide, molecular hydrogen, hydrogen ions and destabilization of metal oxide films. In addition, microbial degradation of crude oil can lead to increased acidity in the oil phase, and oil containing acids is a problem concerning corrosion of pipelines. The reported results showed that the interaction of IB, SRB and TGB accelerated the corrosion rate, and the corrosion in the mixture of IB, SRB and TGB was more serious than in a single microbial system. If this is the case, different treatment system to inhibit corrosion should be considered, among which bactericide agent has received the greatest acceptance. Currently, oxidizer, aldehyde, quatemary ammonium salt and heterocycle compounds has been used as bactericide agents, and Cl_2_, ClO_2_, formaldehyde, pentane-1, 5-dial, trichloroisocyanuric acid (TCCA) and ect [18], but the toxicity tests have been conducted on a limited selection.

In this work, isatin and amino compounds condensed to form the new C = N bond, and it is the isostere of C = O in the structure of isatin, which may ensure the bioactivity of these derivatives similar to isatin. The antifungal activity of these compounds against oilfield microorganism was tested under the concentration of 0.20 g/L and 0.02 g/L, and the results were summarized in Table 4.

**Table 4:** The antifungal activity of isatin derivatives against MIC

**Compound**	**Concentration**	**Microbiotic concentration /mL**		
		**SIB**	**IB**	**TGB**
—	—	110.0	110.0	110.0
1	0.20 g/L	2.5	25.0	70.0
	0.02 g/L	2.5	25.0	110.0
2	0.20 g/L	0.9	6.0	70.0
	0.02 g/L	0.9	2.0	110.0
3	0.20 g/L	0.5	2.5	13.0
	0.02 g/L	0.6	2.0	110.0
4	0.20 g/L	0.6	13.0	13.0
	0.02 g/L	0.0	70.0	70.0
5	0.20 g/L	0.6	110.0	110.0
	0.02 g/L	0.0	110.0	110.0
6	0.20 g/L	0.5	25.0	110.0
	0.02 g/L	0.9	25.0	110.0
7	0.20 g/L	0.0	25.0	2.5
	0.02 g/L	0.6	25.0	2.5
8	0.20 g/L	0.0	110.0	70.0
	0.02 g/L	0.0	110.0	110.0
9	0.20 g/L	0.0	6.0	110.0
	0.02 g/L	0.0	13.0	110.0
10	0.20 g/L	0.0	0.5	13.0
	0.02 g/L	0.6	0.9	110.0

From the table, it can be found that isatin is antifungal active against SRB, but inactive against IB and TGB under both concentrations. For the 5-substitued isatin, compound 2 and 3, the antifungal active against SRB is similar to isatin, slightly more potent against IB, but both, as well as isatin, are inactive against TGB. From the results of the 3-imine indole-2-one (compound 4–10), it was found that the SRB inhibitions are more effective potent under both concentration. While only compound 10 is active against IB with the microbial concentration of 0.5 /mL under the concentration of 0.20 g/L and 0.9 /mL under the concentration of 0.02 g/L. Only compound 7 is active against TGB with the microbial concentration of 2.5 /mL under both concentration.

## Competing interests

The authors declare that they have no competing interests.

## Authors' contributions

GC has conceived the study, formulated the research idea and prepared the manuscript draft version, HS, MZ and FH carried out the chemical synthesis, JZ carried out the Microbiological monitoring, and XH and JZ participated in its design and coordination. All authors have read and approved the final manuscript.
